# Functional analysis of MMR gene VUS from potential Lynch syndrome patients

**DOI:** 10.1371/journal.pone.0304141

**Published:** 2024-06-06

**Authors:** Marwa Mahdouani, Drenushe Zhuri, Hazal Sezginer Guler, Dorra Hmida, Mokni Sana, Mohamed Azaza, Mariem Ben Said, Saber Masmoudi, Fahmi Hmila, Sabri Youssef, Rihab Ben Sghaier, Angela Brieger, Stefan Zeuzem, Ali Saad, Hakan Gurkan, Sinem Yalcintepe, Moez Gribaa, Guido Plotz

**Affiliations:** 1 Laboratory of Cytogenetics, Molecular Genetics and Human Reproduction Biology, Farhat Hached University Hospital, Sousse, Tunisia; 2 Higher Institute of Biotechnology of Monastir, University of Monastir, Monastir, Tunisia; 3 Department of Medical Genetics, Trakya University School of Medicine, Edirne, Turkey; 4 Faculty of Medicine Ibn El Jazzar of Sousse, University of Sousse, Sousse, Tunisia; 5 Department of Dermatology and Venerology, Farhat Hached University Hospital, Sousse, Tunisia; 6 Department of General Surgery, Sahloul University Hospital, Sousse, Tunisia; 7 Laboratory of Molecular and Cellular Screening Processes, Center of Biotechnology of Sfax, Sfax, Tunisia; 8 Department of General and Digestive Surgery, Farhat Hached University Hospital, Sousse, Tunisia; 9 Department of General Surgery, Farhat Hached University Hospital, Sousse, Tunisia; 10 Biomedical Research Laboratory, Medical Clinic 1, University Hospital, Goethe University Frankfurt, Frankfurt am Main, Germany; CNR, ITALY

## Abstract

Lynch syndrome is caused by inactivating variants in DNA mismatch repair genes, namely *MLH1*, *MSH2*, *MSH6* and *PMS2*. We have investigated five *MLH1* and one *MSH2* variants that we have identified in Turkish and Tunisian colorectal cancer patients. These variants comprised two small deletions causing frameshifts resulting in premature stops which could be classified pathogenic (MLH1 p.(His727Profs*57) and MSH2 p.(Thr788Asnfs*11)), but also two missense variants (MLH1 p.(Asn338Ser) and p.(Gly181Ser)) and two small, in-frame deletion variants (p.(Val647-Leu650del) and p.(Lys678_Cys680del)). For such small coding genetic variants, it is unclear if they are inactivating or not. We here provide clinical description of the variant carriers and their families, and we performed biochemical laboratory testing on the variant proteins to test if their stability or their MMR activity are compromised. Subsequently, we compared the results to *in-silico* predictions on structure and conservation. We demonstrate that neither missense alteration affected function, while both deletion variants caused a dramatic instability of the MLH1 protein, resulting in MMR deficiency. These results were consistent with the structural analyses that were performed. The study shows that knowledge of protein function may provide molecular explanations of results obtained with functional biochemical testing and can thereby, in conjunction with clinical information, elevate the evidential value and facilitate clinical management in affected families.

## Introduction

Colorectal cancer accounts for about 10% of all cancers diagnosed each year and cancer-related deaths worldwide [1]. It is the second most common type of cancer diagnosed in women and the third most common type of cancer in men. The global incidence of colorectal cancer is expected to rise to 2.5 million new cases by 2035 [2].

It has a prevalence of approximately 7 per 100.000 people in Turkey, with approximately 5000 new cases and 3200 deaths each year [3]. It is also a serious public health issue in Tunisia, according to the International Agency for Research on Cancer. The ASR (Age Standardized incidence Rate) was 10.9 per 100.000 in 2012, which is a low to medium rate [4]. The predicted CRC ASR would be 39.3/100,000 [CI 95%: 32,9/100,000–48,8/100,000] in 2024 [4].

Lynch syndrome (LS) (OMIM 120435) is the most common hereditary form of CRC, accounting for 3% of all CRC cases [5]. It is caused by a dominantly inherited, inactivating variant in one of four genes involved in post-replicative DNA Mismatch Repair (MMR), namely *MHL1* (OMIM 120436), *MSH2* (OMIM 609309), *MSH6* (OMIM 600678), or *PMS2* (OMIM 600259) [6]. Furthermore, deletions in *EPCAM* (OMIM 185535) have been linked to hypermethylation of the *MSH2* promoter and subsequent *MSH2* silencing [7].

Somatic loss of the WT allele of one of the MMR genes causes cellular deficiency of the affected protein, which can be detected by immune-histochemistry of the tumor tissue. It results in MMR deficiency, resulting in the molecular tumor phenotype of microsatellite instability (MSI) which can be detected by PCR of tumor tissue. The replicative DNA polymerase is unable to correct errors which is thought to be a critical mechanism implicated in the development of Lynch-associated cancers by causing a spontaneous "mutator phenotype" in affected cells [8].

The MSI phenotype is not limited to Lynch syndrome, and can be found in approximately 15% of sporadic colorectal cancers, which most frequently show somatic loss of MLH1 due to promoter hyper-methylation, accompanied by a *BRAF* V600E variant [9].

Lynch syndrome patients are at an increased risk to develop cancers other than CRC such as endometrial cancer, cancers of the small bowel, stomach, ovaries, renal pelvis, ureter, and hepatobiliary system [9].

The majority of germline variants are detected in the *MLH1* gene (50%) followed by the *MSH2* gene (40%), with only 10% found in the *MSH6* and *PMS2* genes [10]. A high proportion of these are of unknown clinical significance [11, 12]. They are termed variants of unclear significance (VUS) or unclassified variants (UV). For properly targeted cancer surveillance in carrier families, the variants require classification, and they must be classified as pathogenic [13]. Without classification as pathogenic, diagnosis cannot be made, and relatives of patients are unable to receive predictive testing and targeted preventive surveillance. Thus, determining the pathogenicity of these increasingly common variants in cancer-predisposing genes presents a significant challenge to clinical geneticists. It is critical to determine which variants in the MMR genes are involved in pathogenesis [14].

The Variant Interpretation Committee (VIC) of the International Society for Gastrointestinal Hereditary Tumors (InSiGHT) used standards established by the International Agency for Research on Cancer (IARC) and has evaluated qualitative or quantitative integration of evidence to classify variants (https://www.insight-group.org/) [15, 16]. The VIC has reclassified some MMR gene VUS as clinically pathogenic (class 5, with probability of pathogenicity >0.99; or class 4, with probability of pathogenicity >0.95) or clinically benign (class 1, with probability of pathogenicity <0.001); or class 2, with probability of pathogenicity <0.05) [15] with clinical recommendations [16, 17].

Segregation analysis in families, population allele frequencies, and tumor pathology are all common methods for analyzing variants in MMR and other cancer predisposition-associated genes, but frequently do not provide sufficient evidential value for a classification. Therefore, functional analysis provides a valuable additional tool to facilitate classification 14]. Structural analysis may further corroborate the conclusions deduced from the functional analysis, specifically if discreet functions can be associated with the analyzed residues [14, 18, 19].

To improve Lynch syndrome diagnosis in our patients, we performed *MLH1* functional analysis for the first time for variants identified in Turkish patients, similar as before for Tunisian patients [14], using a clinically calibrated assay [19].

We investigated four *MLH1* variations, two of which are missense and two are in-frame deletions of three and four residues, found in colorectal cancer patients. We show that the results of these functional analyses are consistent with data obtained from residue conservation and protein structure analysis. This information, when combined with the clinical data provided for the carrier patients, suggests that two variants are likely causative for Lynch syndrome and two others are likely benign. The provided information and results will facilitate more definite classification of the investigated variants, thereby predictive testing for family members and better targeted surveillance measures and lifestyle counseling in affected patients in Tunisia, Turkey and elsewhere [20].

## Materials and methods

### Patients

This study includes both unpublished and published data from three Turkish and three Tunisian patients who met the Amsterdam or the Bethesda criteria. In these patients, germline DNA sequencing was performed, as well as tumor testing (microsatellite instability analysis and immunohistochemistry as indicated). Family cancer histories were obtained [21]. Samples and clinical data were anonymized prior to analysis. The data were accessed for research purposes for Tunisian patients on May 21st, 2021 and for Turkish Patients on June 5th, 2022.

The study was carried out in accordance with the Helsinki Declaration, and it was approved by the Ethics and Research Committee of Farhat Hached University Hospital, Sousse, Tunisia on May 10th, 2021 (OHRP IRB 00008931). The Ethical Committee of Trakya University Faculty of Medicine, Edirne, Turkey declared that its approval was not required for this study. During genetic counseling sessions, all patients were informed about their inclusion in the registries, and written informed consent was obtained from all participants.

### Next generation Sequencing (NGS)

Turkish patients have been sequenced in the medical genetics department of Trakya University Hospital in Turkey using the TruSight^®^ Cancer Sequencing Panel (Illumina) and Qiaseq Targeted DNA Panel (Qiagen) according to the manufacturers’ instructions for NGS. The pooled and barcoded libraries were subsequently sequenced using NextSeq sequencer (Illumina Inc.) [22].

Two of the Tunisian patients were sequenced in the laboratory of Molecular and Cellular Screening Processes, Center of Biotechnology of Sfax in Tunisia using Miseq sequencer (Illumina). One Tunisian patient was sequenced in the Pathology Department of Leiden University Medical Center (LUMC) in the Netherlands as previously described [21]. Briefly, DNA was sequenced with the Ion Proton System (Life Technologies, Carlsbad, CA, USA) using a custom MMR panel [23]. Libraries were prepared with Ion AmpliSeq^™^ Library Kit 2.0 according to the manufacturer’s protocol. The Proton sequencer generated unaligned BAM which were mapped against the human reference genome (GRCh37/hg19) using the TMAP 5.0.7 software with default parameters (https://github.com/iontorrent/TS) [21].

### Nomenclature and classification of genetic variants

The nomenclature guidelines of the Human Genome Variation Society (HGVS) were used to describe the detected genetic variants [24]. Mutalyzer [25] was used to validate the nomenclature of all variants. The recurrence of the identified variants was determined by interrogating four databases: the Leiden Open Variation Database (LOVD), ClinVar, and the Human Gene Mutation Database (HGMD). The InSiGHT database was used to check for current classifications of the variants [15].

### Cell lines

HEK293T cells (deficient for endogenous MLH1 and PMS2) [26] were kindly provided by Prof. Jiricny, Zurich, Switzerland. Their identity was confirmed by comparing their genomic short tandem repeat (STR) profile from 9 loci to the source HEK293T cell line DSMZ ACC 635, and then by a variable number of tandem repeats (VNTR) profile from the Leibnitz Institute DSMZ-German Collection of Microorganisms and Cell Cultures, Braunschweig, Germany, in 02/2009 and 06/2018. They were also routinely tested for mycoplasma and verified by morphology, growth curve analysis, and expression of proteins (e.g., MLH1 and PMS2).

The cells used in this work were freshly thawed from frozen aliquots of these verified batches. They were grown in Dulbecco’s Modified Eagle Medium (Invitrogen) with 10% fetal calf serum (PAA Laboratories) and 1% penicillin-streptomycin (Sigma) [25].

### Protein expression and quantification

The HEK293T cell line, pcDNA3-MLH1, and pSG5-PMS2 have previously been described [26]. The Q5 Site directed mutagenesis system (New England Biolabs, Frankfurt, Germany) was used to generate missense variants with appropriate primes according to the manufacturer’s protocols. Direct sequencing was used to confirm all of the plasmids that were created [14].

HEK293T cells were transfected as previously described [28]. In brief, at 50–70% confluence, HEK293T cells were transiently transfected in 10 cm round dishes with expression plasmids (1 g/ml, respectively) using 10 l/l of the cationic polymer polyethylenimine (Polysciences, Warrington, PA; stock solution 1 mg/ml). Cells were prepared for confocal laser scanning microscopy or protein extraction after 48 hours [25, 27, 28].

The extracts were examined using SDS-PAGE and immunoblotting with anti-MLH1, G168-728 from BD Biosciences, as well as anti-PMS2, E-19, and anti-beta-Actin, C2 from Santa Cruz Biotechnologies. A Fuji LAS-4000 mini camera and Multi Gauge v3.2 were used to detect and quantify chemiluminescence signals (Immobilon, Millipore) [14].

### Evaluation of expression defects with respect to pathogenicity

Protein expression and quantification were performed in parallel with a neutral control variant with impaired stability (MLH1 p.(Val716Met)) and a pathogenic control variant with severe destabilization (MLH1 p.(Ala681Thr)). When the expression of the variant in question is similar to or lower than that of the pathogenic control variant, there is a clinically pathogenic protein stability defect [28, 29].

### MMR activity

A validated procedure was used to assess the *in vitro* MMR activity of MLH1 variants, yielding clinically meaningful results [28, 30]. Protein extracts were mixed with 35 ng of DNA substrate containing a G-T mismatch and a 83-bp single-strand nick. The DNA substrate was purified and digested with EcoRV and AseI after incubation at 37°C. The restriction fragments were separated in agarose gels and then analyzed using GelDoc XR plus detection and QuantityOne software (Bio-Rad). The repair efficiency (e) was calculated as follows: e = (intensity of repaired substrate bands)/ (intensity of all bands of substrate). The amount of DNA recovered during plasmid purification has no effect on this outcome. Total repair efficiencies typically ranged between 50 and 90%. The repair efficiency of MLH1 variants was calculated as e (relative) = e (variant)/e (wild type) * 100 in comparison to a wild-type protein produced in parallel [14].

### Structural and bioinformatic analyses

An alignment containing >900 non-redundant full-length sequences of eukaryotic MLH1 proteins was generated to assess residue conservation using manually curated BLAST hits obtained by interrogating the human MLH1 protein reference sequence NP 00240.1. Multiple hits from the same organism were reduced to one, and non-MLH1 sequences were identified and removed due to the lack of a highly conserved C-terminal FERC motif [31, 32].

Structural analyses were performed using an updated human MutL (MLH1-PMS2) model based on the structure of human PMS2-NTD [33] and homology models of MLH1-NTD and MLH1-PMS2-CTD [27, 34, 35]. Moreover, a complete model of the MLH1-PMS2 heterodimer constructed using AlphaFold2 was used for the investigations [36]. The figures were created with PyMOL v.1.4.1 (Schrodinger LLC). WebLogo was used to create sequence conservation presentations [37].

## Results

### Clinical characteristics of affected carriers of identified MLH1 and MSH2 variants

Six variants identified in six index patients meeting the Amsterdam II and or the Bethesda II guidelines were chosen for analysis (Table 1) [38, 39].

**Table 1:** Clinical, genetic information and functional variant evaluation of the investigated Turkish and Tunisian patients.

Family (Inclusion criteria)	Pati-ent	Sex/Age at diagnosis (years)	Tumor location	MSI status and results of immunohisto—chemistry	Alteration (gDNA) Alteration (protein) dbSNP identifier (Total frequency)	Previous information^1^^,^^2^ /MAPP+PolyPhen-2 prior probability for pathogenicity	Results of the current protein functional evaluation (supporting evidence)	Classifications (InSiGHT and ACMG criteria)
**TURK-1** (Young age)	PIII.4	Female/54	Colon	Normal expression of MSH2/MSH6/MLH1/ PMS2 proteins	MLH1 c.541G>A p.(Gly181Ser) rs1553651157 (none reported)	Class 3 (uncertain)^1^ Uncertain significance (7)^2^ /0.00	**Functional** (functionally neutral, unconserved)	**Uncertain**/Class 3 (InSiGHT) **VUS** 2 points/Class 3 (ACMG)
**TURK-2** (Amster-dam II)	PIII.6	Female/62	Rectum	Normal expression of MLH1/PMS2 proteins Loss of MSH2 and MSH6 ^3^	MLH1 c.1013A>G p.(Asn338Ser) rs63751467 (0.014%)	Class 3 (uncertain)^1^ Uncertain significanc (12)^2^ Benign (3)^2^ Likely benign (2)^2^ /0.03	**Functional** (functionally neutral, unconserved)	**Uncertain**/Class 3 (InSiGHT) **VUS 1** point/Class 3 (ACMG)
**TUN-1** (Bethesda)	PIII.3	Female/42	Right colon	MSI-H Loss of MLH1 and PMS2	MLH1 c.1940_1951 del TGCCCCCTTTGG p.(Val647_Leu650del)	Class 3 (uncertain)^1^ /-^4^	**Defective** (stability decreased like in pathogenic reference variant, no MMR activity, consistent with role in protein structure and IHC findings	**Uncertain**—Class 3 (for class 4 additional functional assay proving deficiency required) (InSiGHT) **Pathogenic**/Class 5 (ACMG)
	PIII.6	Female/44	Sigmoid colon	MSI-H Loss of MLH1 and PMS2				
**TURK-3** (Bethesda)	PII.3	Female/62	Colon + Endome-trium	Loss of MLH1 and PMS2	MLH1 c.2031_2039del TAAAGAATG p.(Lys678_Cys680del)	Novel alteration- No classification /-^4^	**Defective** (stability decreased like in pathogenic reference variant, no MMR activity, consistent with role in protein structure)	**Uncertain**—Class 3 (for class 4 additional functional assay proving deficiency and additional tumor with MSI/IHC-loss required) (InSiGHT) **Likely pathogenic**/Class 4 (ACMG)
**TUN-3** (Amster-dam I)	PIII.5	Male/26	Right Colon + Caecum	-	MLH1 c.2180 delA p.(His727Profs*57)	Novel alteration- No classification /-^4^	No functional evaluation required	**Pathogenic**/Class 5 (InSiGHT) **VUS**/Class 3 (ACMG)
	PIII.4	Male/33	-	-				
**TUN-2** (Bethesda)	PIII.5	Male/57	Right Colon	-	MSH2 c.2362_2363 insA p.(Thr788Asnfs*11)	Novel alteration- No classification /-^4^	No functional evaluation required	**Pathogenic**/Class 5 (InSiGHT) **Pathogenic**/Class 3 (ACMG)

^1^ Retrieved in January 2024 from the InSiGHT database (http://www.insight-database.org/classifications/)

^2^ Retrieved in January 2024 from the ClinVar database (https://www.ncbi.nlm.nih.gov/clinvar/); numbers in brackets give the number of individual reports

^3^ Sequencing and MLPA of *MSH2* and *MSH6*: normal

^4^ Only for missense alterations, MAPP+PolyPhen-2 values are available.

Three of the variants were found in Tunisian patients. Two of them are novel variants which encode insertions leading to a frameshift and premature stop codons in *MLH1* (c.2180delA, corresponding to p.(His727Profs*57)) and *MSH2* (c.2362_2363insA, corresponding to p.(Thr788Asnfs*11)). These were excluded from further functional analysis in this study since they can be classified as pathogenic without further efforts (see below). The third Tunisian patient carried a small *in-frame* deletion in *MLH1* (c.1940_1951del, corresponding to p.(Val647_Leu650del)) [21]. The clinical significance of this variant is unclear.

In Turkey, we identified three genetic MLH1 variants. Two missense VUS variants (MLH1 c.1013A>G, corresponding to p.(Asn338Ser) and MLH1: c.541G>A, corresponding to p.(Gly181Ser)). We furthermore identified another small, in-frame deletion variant (MLH1: c.2031_2039delTAAAGAATG, corresponding to the three-residue protein deletion p.(Lys678_Cys680del)) that has not been reported before. The p.(Asn338Ser) variant has already been described before in South America [40, 41].

**Fig 1:**
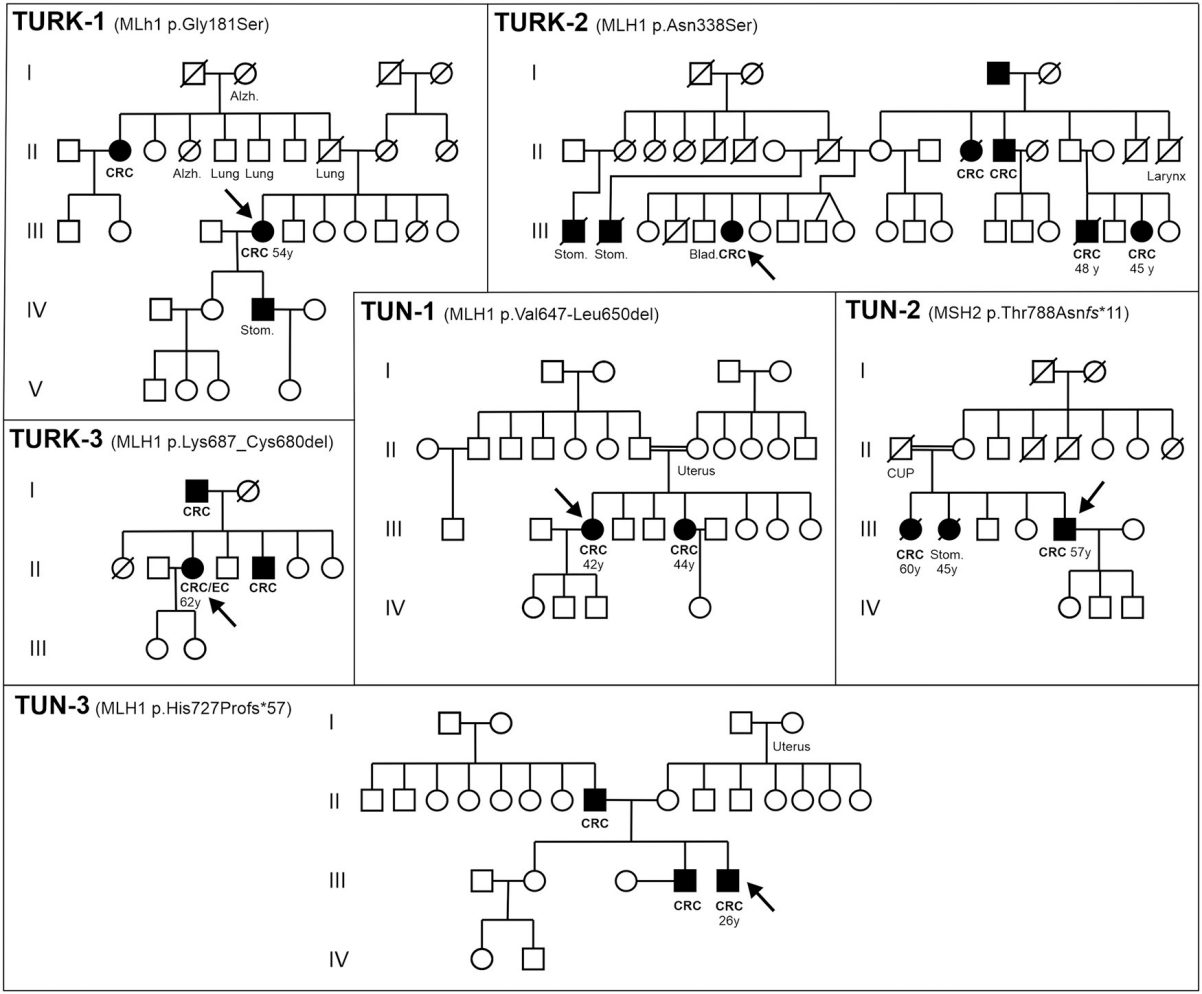
Pedigrees of variant carriers. Arrows show index cases. Double lines indicate consangious marriages. Tumours and other diseases are indicated under the symbols (CRC: colorectal cancer; Stom.: stomach cancer; Blad.: bladder cancer; Lung, Larynx: cancers affecting lung and larynx; CUP: cancer of unknown primary; EC: endometrium cancer; Uterus: uterus cancer; Alzh.: Alzheimer’s disease). Age at diagnosis is indicated where available. Filled symbols indicate tumours elevated in Lynch syndrome.

Family data was acquired for all affected patients (Fig 1). Additional cancer cases were present in all families. Most of the tumors in index cases were colorectal, showing a loss of MLH1 protein expression except for the carrier of variant p.(Gly181Ser) for whom immunohistochemistry showed a normal expression of the MMR proteins (Table 1).

This patient (Family TURK-1, PIII.4) was diagnosed at the age of 54 with colon cancer in stage III. His paternal aunt also suffered from the same type of cancer; his son developed a stomach cancer and other members in his family developed lung cancer.

The variant p.(Asn338Ser) was discovered in family TURK-2, in which 5 members (PII.11, PII.12, PIII.6, PIII.17 and PIII.19) were affected by CRC. DNA sequencing was carried out for only one individual of them (PIII.6), who had rectum cancer at the age of 62. The tumor of this index case showed a normal expression of MLH1 and PMS2 proteins. Two other family members were diagnosed with stomach cancer (PIII.1 and PIII.2), one (PII.17) affected by laryngeal cancer and another one was affected by bladder cancer (PIII.5).

In TURK-3, the index case (PII.3), who is a confirmed carrier of the variant p.(Lys678_Cys680del), had synchronous colon and endometrium cancers. She was diagnosed at the age of 62. Her CRC showed a loss of MLH1 and PMS2 protein expression in immunohistochemistry. Her father and her brother were also diagnosed with colon cancer but they were not tested.

Patients PIII.3 and PIII.6 (two sisters from a consanguineous marriage) of the family TUN-1 were found to carry the same variant, p.(Val647_Leu650del). They developed colorectal cancer at the ages of 42 and 44, respectively. Both showed loss of MLH1 and PMS2 protein expression and loss of heterozygosity with retention of the variant in their tumors. Their paternal cousin also has CRC, while their mother had uterine cancer [21].

The variant p.(Thr788Asnfs*11) was identified in patient (PIII.5) of family TUN-2. This index case suffered from the Muir-Torre syndrome with multiple sebaceous adenomas associated with the development of polyps discovered after analysis of the operating piece of right-colonic carcinoma that was operated at the age of 57 years. His two sisters (PIII.1) and (PIII.2) developed gastric and colonic cancers separately at the age of 45 and 60, respectively. Their father died presumably of a cancer disease.

Finally, in the family TUN-3, we found the variant p.(His727Profs*57) in the index case (PIII.5) who developed a colonic tumor at the age of 26 and underwent a right hemi-colectomy one year later. This variant was also identified in his brother (PIII.4), who was addressed to our center for a genetic counseling. At the time of consultation, he was 33 years old and did not exhibit any symptoms associated with the variant. Most likely, Lynch syndrome was inherited from their father (PII.8) who developed caecum cancer at the age of 46 and underwent a right hemi-colectomy; unfortunately, this patient was not available for genetic testing. This family is positive for the Amsterdam II criteria.

Two of the identified variants can be classified straightforwardly as pathogenic (class 5) by application of the criteria provided by the variant interpretation committee (VIC) of the International Society for Gastroentestinal Hereditary Tumours (InSiGHT) (https://www.insight-group.org/criteria/). Their expected result is a premature translational stop, resulting in a non-functional, truncated protein: MLH1 p.His727Profs*57 (TUN-3) and MSH2 p. p.Thr788Asnfs*11 (TUN-2) (Table 2).

**Table 2:** Classification criteria as applied to the current variants^1^.

Criteria for class 5 (pathogenic)	p.(Thr788Asnfs*11)	p.(His727Profs*57)
Coding sequence variation resulting in a stop codon i.e. a nonsense or a frameshift alteration that is not after codon 743 in *MLH1* or after codon 888 in MSH2, and not in the last exon of *MSH6* or *PMS2*.	+	+
**Criteria for class 4 (likely pathogenic)**	**p.(Lys678_Cys680del)**	**p.(Val647_Leu650del)**
Function lost due to variant confirmed by 2 independent laboratories	+ (this work) 0	+ (this work) 0
+ one of the following		
• co-segregation with disease results in LR of >5:1	0	0
• ≥ 2 families with ≥ 2 affected non-proband carriers	0	0
• ≥ 2 independent tumors with MSI and/or loss of MMR protein expression consistent with the variant location	+ IHC of TURK-3 PII.3 0	+ IHC of TUN-1 PIII.3 + IHC of TUN-1 PIII.6
**Criteria for class 2 (likely neutral)**	**p.(Gly181Ser)**	**p.Asn338Ser)**
Proficient protein function comprising all of the following tests:		
• Proficient in mRNA test	0	+ (Tournier *et al*. 2008) [50]
• Proficient in two independent MMR tests	+ (this work) 0	+ (this work) + (Köger *et al*. 2018)
• Proficient in expression (>75%)	+ (this work)	+ (this work)
• Proficient in subcellular localization	0	0
+ one of the following		
• Present in control group at 0.01–1%	+ (0.019%)^2^	0
• Lack of co-segregation with disease (LR≤0.01)	0	0
• ≥ 3 CRC with MSS or no loss of MMR protein expression	+ IHC of TURK-1 PIII.4 0 0	0 0 0

^1^ referring to the current criteria as documented in the file 2018–06_InSiGHT_VIC_v2.4.pdf available at https://www.insight-group.org/criteria/. This table summarizes only an excerpt of these criteria applicable to the given cases. + means that one criterion is fulfilled, 0 means that for one criterion the data point is lacking. IHC, immunohistochemistry.

^2^ Frequency of the SNP rs63751467 in the European population according to the dbSNP database (retrieved September 2023).

In contrast, the available clinical data are insufficient to classify the small coding variants: it was not possible to perform additional genetic analyses in all relatives of patients to assess co-segregation, which is a highly reliable method for the assessment of pathogenicity. Moreover, no sufficient information on molecular tumor traits (microsatellite instability and BRAF status) was available for pathogenicity clarification.

Consequently, it is unclear if the described small coding variants are pathogenic, and if the carrier patients indeed have Lynch syndrome. For assessing pathogenicity, we therefore set out to assemble further informative knowledge on alternative ways and performed functional testing of the genetic variants *in vitro*.

### Evaluation of protein stability and MMR efficiency of MLH1 variants by functional analysis

We performed functional analyses on the four small coding variants to determine if the identified alterations cause a defect of function in the protein; if this were the case, a causal involvement in the observed cancer cases is most likely.

**Fig 2:**
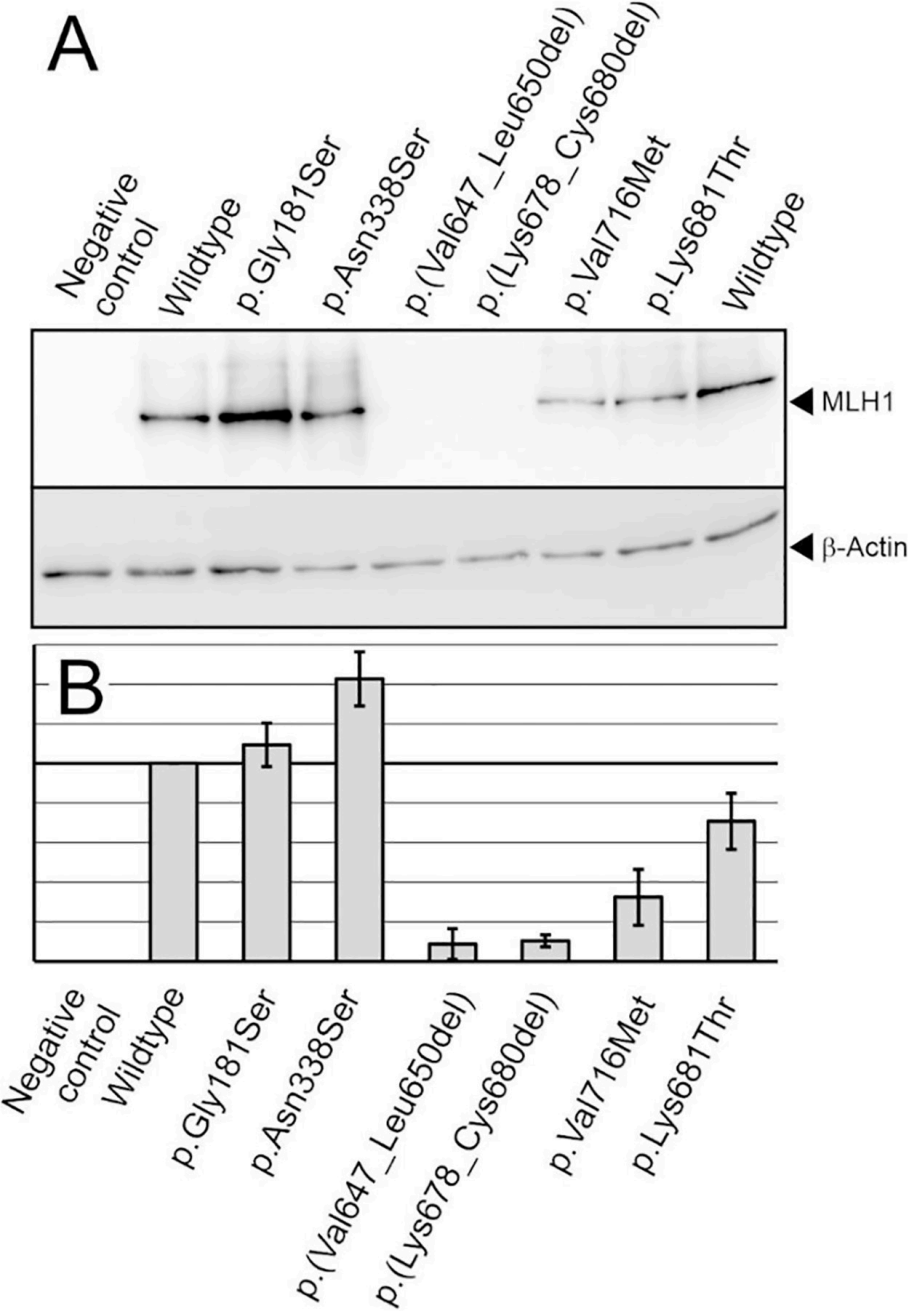
Analysis of expression of the MLH1 variants. **A**, Expression of wild-type and variant MLH1 proteins was visualized by SDS-PAGE and western blotting. The two stability reference variants p.(Ala681Thr) (pathogenic expression defect) and p.(Val716Met) (non-pathogenic expression defect, polymorphism) were transfected in parallel. The shown blots are representative for 3 independent experiments that were performed and delivered the data shown in evaluation (B). **B**, Average expression values in percent of the wild-type expression and standard deviations are shown for wild-type and variant MLH1 proteins.

Small coding variants frequently cause functional defects in MLH1 by destabilizing the protein or by interfering with DNA repair activity [28, 32, 40, 41]. Protein stability decreases lead to lower intracellular protein levels, which, even if the protein is functional, may result in DNA mismatch repair deficiency if it falls below a certain threshold [30]. We used previously established reference variants to translate expression defects into pathogenicity statements [30]. Of these, the MLH1 p.(Ala681Thr) pathogenic Lynch syndrome reference variant served to identify variants whose destabilization is severe enough to confer a pathogenic effect in humans due to low cellular protein levels. Furthermore, the neutral, slightly destablized clinically neutral polymorphism MLH1 p.(Val716Met) was used as a reference for clinically neutral stability defects [30]. We thoroughly compared the expression levels of all variants to the wild-type MLH1 protein and the two reference variants (Fig 2A). The outcomes of several independent experiments were summarized (Fig 2B).

Two variants, p.(Gly181Ser) and p.(Asn338Ser), were as strongly expressed as the wild-type protein and thus do not exhibit clinically relevant stability issues, as evidenced by expression of the p.(Val716Met) reference variant (Fig 2B). The T-test revealed no statistically significant difference between them and the wild-type protein (P>0.05).

In contrast, the variants p.(Lys678_Cys680del) and p.(Val647_Leu650del) showed significantly compromised stability at the limit of detection, the decrease was much stronger than that of the pathogenic reference variant p.(Ala681Thr).

A pathogenic defect can be concluded for these highly destabilized variants because insufficient MLH1 protein is present in the cell [30].

**Fig 3:**
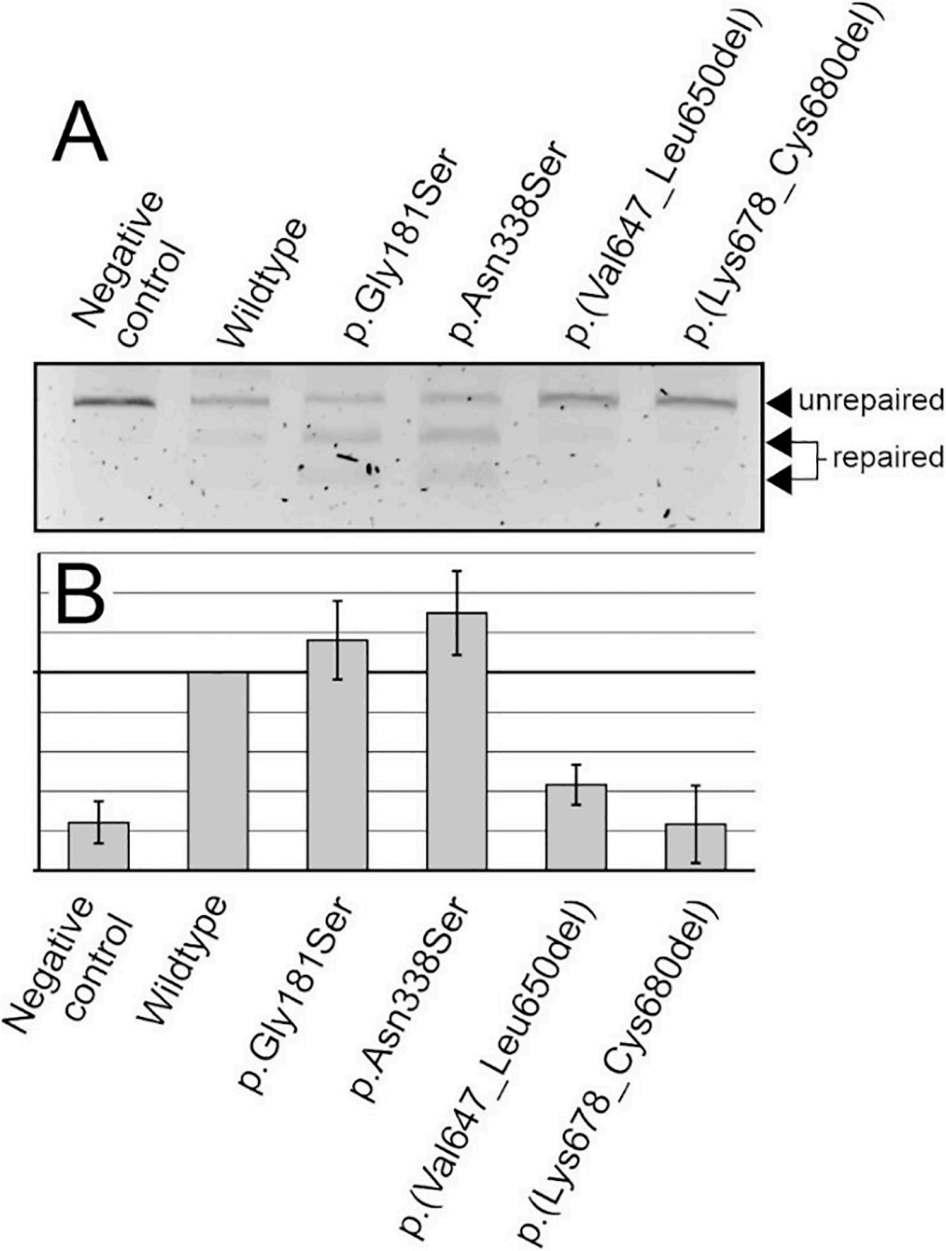
Analysis of mismatch repair activity of the MLH1 variants. DNA mismatch repair activity was assessed for wild-type and variant MLH1 proteins, and a negative control (without MLH1 protein) was included as detailed in “Materials and Methods”. **A**, Representative agarose gel image of the MMR activity measurement. The extent of repair is visible in the agarose gel electrophoresis by the generation of two smaller fragments (“Repair”) of the unrepaired, linearized plasmid (“No repair”). **B**, three independent experiments were performed, and repair activity was scored relative to wild-type MLH1 protein (100%). Average repair values and standard deviations are shown.

Another major reason that small coding MLH1 variants may be pathogenic is that they confer catalytic inactivity of the variant MLH1 protein. Since the primary function of MLH1 is to aid in DNA mismatch repair, we tested the variants’ ability to perform the repair reaction *in vitro* (Fig 3A). Several independent experiments were carried out to validate the findings (Fig 3B).

The p.(Gly181Ser) and p.(Asn338Ser) variants had MMR efficiency comparable to the wildtype, whereas the p.(Lys678_Cys680del) and p.(Val647_Leu650del) variants were approximately similar to the negative control and had therefore completely lost repair activity.

Taken together, in this study, functional assays revealed that p.(Lys678_Cys680del) and p.(Val647_Leu650del) are variants that confer a functional defect on the MLH1 protein. However, the functionality of the variants p.(Gly181Ser) and p.(Asn338Ser) was, in the applied assay systems, indistinguishable from that of wild-type.

### Conservation and structural roles of the affected residues

The analysis of the structural role and the conservation of variant residues can help to explain the findings of functional studies. This can represent confirmatory evidence if the gathered information is in good agreement with the functional observations. We therefore evaluated the conservation and positions of the affected residues within the structure of the MLH1-PMS2 heterodimer.

**Fig 4:**
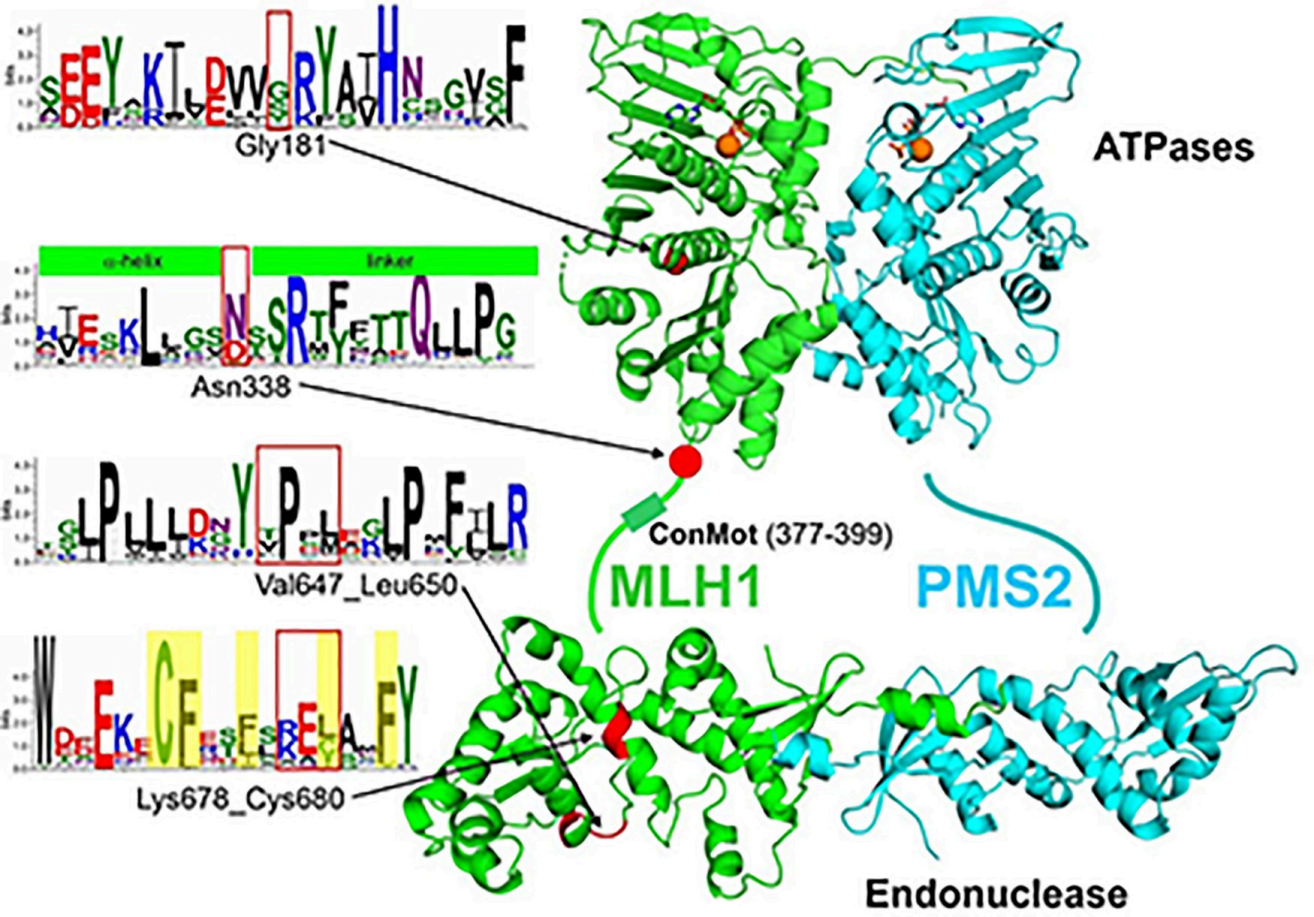
Affected residues in their structural and conservational context. Structural model of the human MLH1-PMS2 heterodimer (MLH1: green; PMS2: cyan). The N-terminal, structured ATPase regions (bound ATP in ball-stick presentation with magnesium ions as orange spheres) are connected by unstructured linker region (symbolized as a line), including the catalytically relevant, conserved ConMot motif, to the structured C-terminal dimerization and endonuclease domains. The locations of the residues affected by alterations investigated in this study are marked in red in the structure and by red boxes in the sequence logos. Sequence conservation is shown in WebLogo presentation at the left. Yellow shading indicates hydrophobic residues relevant for helix anchoring. Green boxes above the sequence inform about secondary structure that these sequences form.

Gly181 is located in the N-terminal ATPase domain, Arg338 is placed in the unstructured linker, and the two small deletions ΔVal647_Leu650 and ΔLys678_Cys680 are both found in the C-terminal dimerization domain of MLH1 (Fig 4).

The glycine at codon 181 is replaced by serine, an amino acid with similar biochemical properties. Gly181 is located at the backside β-sheet of the ATPase pocket, but directed outwards and not involved in ATP binding (Fig 4, right panel). The residue is poorly conserved in eukaryotic species (Fig 4, left panel), suggesting that substitutions are of little effect on protein function and stability. Moreover, Gly181 is frequently substituted by serine in eukaryotic organisms (Fig 4, left panel). All these observations strongly suggest that Gly181Ser is a neutral substitution, which is in perfect agreement with the results of the biochemical analyses.

Asn338 is located at the very beginning (first residue) of the unstructured linker that connects the N- and C-terminal domains (Fig 4). The linker is in the largest part neither conserved nor structured and rarely contains pathogenic alterations [42]. An exception is represented by a small, functionally highly relevant motif which is in sufficient distance to Asn338 (in Fig 4 depicted as ConMot) [18]. The linker also shows some conservation in the shown N-terminal region following the α-helix and Asn338 (Fig 4, left panel). For example, there is a strongly conserved arginine, which is a frequently observation at the C-termini of α-helices. They are often involved in formation of helix capping by forming loops through main chain interactions and compensating helix dipols [43], which is also the case here according to AlphaFold2 structure predictions. The Asn338 itself shows only intermediate conservation (Fig 4, left panel: N). Besides asparagine (N), aspartate (D) but also serine (S) occur regularly in MLH1 proteins [32], suggesting that it is a tolerated substitution. Additionally, no specific function of the Asn338 residue is obvious from structural analyses. This is in agreement with the biochemical finding that Asn338Ser has a normal protein function like wildtype.

In comparison to missense substitutions, deletions can be expected to confer more dramatic effects on average, since they have a greater potential to distort the structure of a protein. However, if deletions or insertions are located in unstructured areas (intrinsically disordered regions, IDRs), or loops between secondary structural elements, they may also be tolerated and not confer any effect. This we have described before for extensive artificial deletions in the MLH1 linker [18] but also for a small deletion identified in a human cancer patient that is located at the border between a C-terminal helix and a loop [31].

The deletion Val647_Leu650 (VPPL) affects the beginning of an α-helix including a fraction of the preceding loop and comprises the strongly conserved Pro648 residue (Fig 4). This highly conserved proline likely facilitates helix formation and orientation, since it is placed in a typical position [31]. The deletion removes this relevant proline and significantly shortens the loop structure required for correct positioning of the helix, thereby destroying the local structure of the MLH1 protein, explaining the instability and the biochemical loss of function.

The deletion Lys678_Cys680 affects the middle of an extended α-helix. While none of the three deleted residues displays significant conservation, the most dramatic effect is conferred by the relative rotation of the conserved hydrophobic residues of the helix (marked yellow in Fig 4). These hydrophobic residues are normally oriented towards one side of the helix, pointing to the proteins’ interior and serve to anchor this side of the helix in the hydrophobic core of the protein. As a result of the deletion, the orientation of internal (hydrophobic) and external (hydrophilic) residues is distorted (S1 Fig). Consequently, it can be expected to have a significant impact on protein structure (and function), as evidenced by the low stability of the variant protein.

Thus, on summary, there is a high consistency of the functional results and the information deduced from residue conservation and role in protein structure.

## Discussion

In this study, we report identification, description and evaluation of five MLH1 and one MSH2 variants found in three Turkish and four Tunisian patients with suspected Lynch syndrome. The identified variants have either not been reported before or have no informative classification of their clinical effects, forestalling predictive testing and targeted surveillance in affected families (Table 1). Like in many similar cases, even when summarizing the available evidence from our current investigation and others, it remains unclear if these protein variants are functional, and clinical data is insufficient for pathogenicity classification. We have therefore performed functional and structural analyses for providing additional lines of evidence concerning their biological and clinical effects. For functional investigation, we utilized an standardized assay [14] that uses clinically established reference variants for pathogenicity assessment and [14, 44]. While the methodologically similar CIMRA assay directly enables calculating of posterior probabilities of pathogenicity [45], this procedure provides better data on the molecular reasons of functional loss.

Of the examined variants, two variants could be classified straightforwardly as pathogenic (class 5) by application of the criteria provided by the variant interpretation committee (VIC) of the International Society for Gastrointestinal Hereditary Tumours (InSiGHT) (https://www.insight-group.org/criteria/), since their expected result is a premature translational stop, resulting in a truncated, non-functional protein (Table 2). The ACMG classification [46] is concordant for the MSH2 truncating variant which also includes expert panel reviews (S1 Table) [15, 47–49]. However, the ACMG classification scheme is more cautious for the MLH1 truncating variant since the truncation removes only a comparatively small C-terminal fraction of the protein (S1 Table). However, this fraction contains important, highly conserved functional sequence from the MLH1 protein indispensable for function, and truncations starting from position MLH1 Tyr750 have been shown to be deleterious before [18, 19]. Consistent with this, the affected family (TUN-3) fulfills the Amsterdam II criteria (Fig 1).

Concerning the two other missense VUS variants and two in-frame alterations, one is novel and the other three are currently classified as variants of uncertain significance (Table 1).

For the two deletion variants, we could show loss of expression compatible with a disease-causing defect using a clinically calibrated MMR assay system [30]. All results of this assay system have yet been consistent with variant classifications where available (see S1 Table of [14]), confirming its reliability. Moreover, both deletions destroy secondary structures, and they are located within a domain for which we have shown before that alterations make the protein susceptible to destabilization [30]. This is well consistent with the absence of MLH1 in the patients’ tumors in IHC (Table 1). Both variants currently data lack for a final classification in class 4 (likely pathogenic) according to current InSiGHT criteria for variant classification (Table 2). However, the AMCG guidelines allow the conclusion that the variants are pathogenic (Table 1, S1 Table).

The findings for the missense variants (p.(Gly181Ser) and p.(Asn338Ser)) supported neutrality: there were no detectable defects in functionality. These variants retained a sufficiently high expression level that is above the clinical reference variant for proficient stability (p.(Val716Met)), and catalytic activity was not compromised by the alterations. These observations are consistent with the low conservation of the residues and the occurrence of the substitutions in evolution, and also consistent with their very low MAPP+PolyPhen-2 prior probability of pathogenicity (Table 1). However, providing functional evidence for neutrality is more complex since it requires to exclude all potential sources of defects. Both variants were proficient in the applied investigations which comprise the most relevant reasons underlying deficiency caused by missense alterations, namely stability and repair activity [30, 31]; moreover, sufficient complimentary clinical data are also required according to the InSiGHT criteria but currently insufficient (Table 2). Application of the ACMG guidelines therefore also currently results in uncertain significance (Table 1, S1 Table).

The finding that the tumor of the index patient showed normal expression of MLH1 and PMS2 is consistent with the normal expression of the p.Asn338Ser variant; it is noteworthy that this tumor displayed loss of MSH2 and MSH6 expression, which would be compatible with an inactivating variant in the *MSH2* gene. However, sequencing of the *MSH2* gene did not reveal any variants, and MLPA analysis also did not detect any deletions or duplications in this gene.

Taken together, all functional findings suggest that both variant proteins are proficient. This is consistent with the clinical observation that there does not seem to be a predisposition in family TURK-1. In contrast, we did not find evidence that the potential cancer predisposition observed in family TURK-2 (with positive Amsterdam II criteria) is caused by their variant. However, there remains a small possibility that these variants may interfere with other functional aspects of the MLH1 gene, e. g. with mRNA formation or DNA damage response.

In conclusion, at a time when Next-Generation Sequencing may enable the detection of novel and multiple VUS in distinct cancer genes, functional characterization of these VUS will become more important in genetic counseling for these families.

Biochemical function analysis, performed with appropriate controls allowing a clinically meaningful reading of the results, in conjunction with additional evidence, is able to provide a reliable statement of pathogenicity if consistent results are obtained. This approach can therefore fill the gap if other methods of classification do not offer sufficient probative value.

To our knowledge, we have performed for the first time functional testing for unclear MLH1 genetic variants identified in Turkish cancer patients.

## Supporting information

S1 Fig Structure detail of the deletion p.Lys678_Cys680del. (TIF)

S1 Table ACMG classification for the MMR variants (guidelines 2015). (DOCX)

S1 File (DOCX)

## References

[1] Bray F, Ferlay J, Soerjomataram I, Siegel RL, Torre LA, Jemal A. Global cancer statistics 2018: GLOBOCAN estimates of incidence and mortality worldwide for 36 cancers in 185 countries. CA Cancer J Clin. nov 2018;68(6):394‑424. doi: 10.3322/caac.21492 30207593

[2] Arnold M, Sierra MS, Laversanne M, Soerjomataram I, Jemal A, Bray F. Global patterns and trends in colorectal cancer incidence and mortality. Gut. avr 2017;66(4):683‑91. doi: 10.1136/gutjnl-2015-310912 26818619

[3] Tatar M, Tatar F. Colorectal cancer in Turkey: current situation and challenges for the future. Eur J Health Econ HEPAC Health Econ Prev Care. janv 2010;10 Suppl 1:S99–105. doi: 10.1007/s10198-009-0197-7 20012668

[4] Khiari H, Ben Ayoub HW, Ben Khadhra H, Hsairi M. Colorectal Cancer Incidence Trend and Projections in Tunisia (1994–2024). Asian Pac J Cancer Prev APJCP. 26 oct 2017;18(10):2733‑9. 29072401 10.22034/APJCP.2017.18.10.2733 PMC5747397

[5] Biller LH, Syngal S, Yurgelun MB. Recent advances in Lynch syndrome. Fam Cancer. avr 2019;18(2):211‑9. doi: 10.1007/s10689-018-00117-1 30627969 PMC6450737

[6] Lynch HT, Snyder CL, Shaw TG, Heinen CD, Hitchins MP. Milestones of Lynch syndrome: 1895–2015. Nat Rev Cancer. mars 2015;15(3):181‑94. doi: 10.1038/nrc3878 25673086

[7] Ligtenberg MJL, Kuiper RP, Chan TL, Goossens M, Hebeda KM, Voorendt M, et al. Heritable somatic methylation and inactivation of MSH2 in families with Lynch syndrome due to deletion of the 3’ exons of TACSTD1. Nat Genet. janv 2009;41(1):112‑7. doi: 10.1038/ng.283 19098912

[8] Drost M, Tiersma Y, Thompson BA, Frederiksen JH, Keijzers G, Glubb D, et al. A functional assay-based procedure to classify mismatch repair gene variants in Lynch syndrome. Genet Med Off J Am Coll Med Genet. juill 2019;21(7):1486‑96. doi: 10.1038/s41436-018-0372-2 30504929 PMC7901556

[9] Dekker E, Tanis PJ, Vleugels JLA, Kasi PM, Wallace MB. Colorectal cancer. Lancet Lond Engl. 19 oct 2019;394(10207):1467‑80. 10.1016/S0140-6736(19)32319-0 31631858

[10] Peltomäki P. Deficient DNA mismatch repair: a common etiologic factor for colon cancer. Hum Mol Genet. avr 2001;10(7):735‑40. doi: 10.1093/hmg/10.7.735 11257106

[11] Liccardo R, Lambiase M, Nolano A, De Rosa M, Izzo P, Duraturo F. Significance of rare variants in genes involved in the pathogenesis of Lynch syndrome. Int J Mol Med. juin 2022;49(6):81. doi: 10.3892/ijmm.2022.5137 35475445 PMC9083887

[12] Peltomäki P, Vasen H. Mutations associated with HNPCC predisposition—Update of ICG-HNPCC/INSiGHT mutation database. Dis Markers. 2004;20(4‑5):269‑76. doi: 10.1155/2004/305058 15528792 PMC3839397

[13] Tavtigian SV, Greenblatt MS, Goldgar DE, Boffetta P, IARC Unclassified Genetic Variants Working Group. Assessing pathogenicity: overview of results from the IARC Unclassified Genetic Variants Working Group. Hum Mutat. nov 2008;29(11):1261‑4. 18951436 10.1002/humu.20903 PMC2966307

[14] Mahdouani M, Ben Ahmed S, Hmila F, Rais H, Ben Sghaier R, Saad H, et al. Functional characterization of MLH1 missense variants unveils mechanisms of pathogenicity and clarifies role in cancer. PloS One. 2022;17(12):e0278283. doi: 10.1371/journal.pone.0278283 36454741 PMC9714755

[15] Thompson BA, Spurdle AB, Plazzer JP, Greenblatt MS, Akagi K, Al-Mulla F, et al. Application of a 5-tiered scheme for standardized classification of 2,360 unique mismatch repair gene variants in the InSiGHT locus-specific database. Nat Genet. févr 2014;46(2):107‑15. doi: 10.1038/ng.2854 24362816 PMC4294709

[16] Plon SE, Eccles DM, Easton D, Foulkes WD, Genuardi M, Greenblatt MS, et al. Sequence variant classification and reporting: recommendations for improving the interpretation of cancer susceptibility genetic test results. Hum Mutat. nov 2008;29(11):1282‑91. doi: 10.1002/humu.20880 18951446 PMC3075918

[17] Thompson BA, Greenblatt MS, Vallee MP, Herkert JC, Tessereau C, Young EL, et al. Calibration of multiple in silico tools for predicting pathogenicity of mismatch repair gene missense substitutions. Hum Mutat. janv 2013;34(1):255‑65. doi: 10.1002/humu.22214 22949387 PMC4318556

[18] Wolf K, Kosinski J, Gibson TJ, Wesch N, Dötsch V, Genuardi M, et al. A conserved motif in the disordered linker of human MLH1 is vital for DNA mismatch repair and its function is diminished by a cancer family mutation. Nucleic Acids Res. 7 juill 2023;51(12):6307‑20. doi: 10.1093/nar/gkad418 37224528 PMC10325900

[19] Identification of Lynch syndrome mutations in the MLH1-PMS2 interface that disturb dimerization and mismatch repair—PubMed [Internet]. [cité 10 janv 2023]. Disponible sur: https://pubmed.ncbi.nlm.nih.gov/20533529/10.1002/humu.21301 PMC2908215 20533529

[20] Sievänen T, Törmäkangas T, Laakkonen EK, Mecklin JP, Pylvänäinen K, Seppälä TT, et al. Body Weight, Physical Activity, and Risk of Cancer in Lynch Syndrome. Cancers. 13 avr 2021;13(8):1849. 33924417 10.3390/cancers13081849 PMC8069994

[21] Ben Sghaier R, Jansen AML, Bdioui A, Van Wezel T, Ksiaa M, Elgolli L, et al. Targeted next generation sequencing screening of Lynch syndrome in Tunisian population. Fam Cancer. juill 2019;18(3):343‑8. doi: 10.1007/s10689-019-00130-y 31114938

[22] Yalcintepe S, Gurkan H, Demir S, Tozkir H, Tezel HA, Atli EI, et al. Targeted next-generation sequencing as a diagnostic tool in gastrointestinal system cancer/polyposis patients. Tumori. déc 2020;106(6):510‑7. doi: 10.1177/0300891620919171 32390558

[23] Jansen AM, van Wezel T, van den Akker BE, Ventayol Garcia M, Ruano D, Tops CM, et al. Combined mismatch repair and POLE/POLD1 defects explain unresolved suspected Lynch syndrome cancers. Eur J Hum Genet EJHG. juill 2016;24(7):1089‑92. doi: 10.1038/ejhg.2015.252 26648449 PMC5070903

[24] den Dunnen JT, Antonarakis SE. Mutation nomenclature extensions and suggestions to describe complex mutations: a discussion. Hum Mutat. 2000;15(1):7‑12. doi: 10.1002/(SICI)1098-1004(200001)15:1&lt;7::AID-HUMU4&gt;3.0.CO;2-N 10612815

[25] Wildeman M, van Ophuizen E, den Dunnen JT, Taschner PEM. Improving sequence variant descriptions in mutation databases and literature using the Mutalyzer sequence variation nomenclature checker. Hum Mutat. janv 2008;29(1):6‑13. doi: 10.1002/humu.20654 18000842

[26] Trojan J, Zeuzem S, Randolph A, Hemmerle C, Brieger A, Raedle J, et al. Functional analysis of hMLH1 variants and HNPCC-related mutations using a human expression system. Gastroenterology. janv 2002;122(1):211‑9. doi: 10.1053/gast.2002.30296 11781295

[27] Borràs E, Pineda M, Brieger A, Hinrichsen I, Gómez C, Navarro M, et al. Comprehensive functional assessment of MLH1 variants of unknown significance. Hum Mutat. nov 2012;33(11):1576‑88. doi: 10.1002/humu.22142 22736432

[28] Brieger A, Adam R, Passmann S, Plotz G, Zeuzem S, Trojan J. A CRM1-dependent nuclear export pathway is involved in the regulation of MutLα subcellular localization. Genes Chromosomes Cancer. févr 2011;50(2):59‑70. 21064154 10.1002/gcc.20832

[29] Plotz G, Welsch C, Giron-Monzon L, Friedhoff P, Albrecht M, Piiper A, et al. Mutations in the MutSalpha interaction interface of MLH1 can abolish DNA mismatch repair. Nucleic Acids Res. 2006;34(22):6574‑86. doi: 10.1093/nar/gkl944 17135187 PMC1747184

[30] Hinrichsen I, Brieger A, Trojan J, Zeuzem S, Nilbert M, Plotz G. Expression defect size among unclassified MLH1 variants determines pathogenicity in Lynch syndrome diagnosis. Clin Cancer Res Off J Am Assoc Cancer Res. 1 mai 2013;19(9):2432‑41. doi: 10.1158/1078-0432.CCR-12-3299 23403630

[31] Hinrichsen I, Schäfer D, Langer D, Köger N, Wittmann M, Aretz S, et al. Functional testing strategy for coding genetic variants of unclear significance in MLH1 in Lynch syndrome diagnosis. Carcinogenesis. févr 2015;36(2):202‑11. doi: 10.1093/carcin/bgu239 25477341

[32] Köger N, Paulsen L, López-Kostner F, Della Valle A, Vaccaro CA, Palmero EI, et al. Evaluation of MLH1 variants of unclear significance. Genes Chromosomes Cancer. juill 2018;57(7):350‑8. doi: 10.1002/gcc.22536 29520894

[33] Bustin SA, Benes V, Garson JA, Hellemans J, Huggett J, Kubista M, et al. The MIQE guidelines: minimum information for publication of quantitative real-time PCR experiments. Clin Chem. avr 2009;55(4):611‑22. doi: 10.1373/clinchem.2008.112797 19246619

[34] Smith CE, Mendillo ML, Bowen N, Hombauer H, Campbell CS, Desai A, et al. Dominant mutations in S. cerevisiae PMS1 identify the Mlh1-Pms1 endonuclease active site and an exonuclease 1-independent mismatch repair pathway. PLoS Genet. oct 2013;9(10):e1003869. doi: 10.1371/journal.pgen.1003869 24204293 PMC3814310

[35] Gueneau E, Dherin C, Legrand P, Tellier-Lebegue C, Gilquin B, Bonnesoeur P, et al. Structure of the MutLα C-terminal domain reveals how Mlh1 contributes to Pms1 endonuclease site. Nat Struct Mol Biol. avr 2013;20(4):461‑8. 23435383 10.1038/nsmb.2511

[36] Guarné A, Junop MS, Yang W. Structure and function of the N-terminal 40 kDa fragment of human PMS2: a monomeric GHL ATPase. EMBO J. 1 oct 2001;20(19):5521‑31. doi: 10.1093/emboj/20.19.5521 11574484 PMC125661

[37] Kosinski J, Plotz G, Guarné A, Bujnicki JM, Friedhoff P. The PMS2 subunit of human MutLalpha contains a metal ion binding domain of the iron-dependent repressor protein family. J Mol Biol. 10 oct 2008;382(3):610–27. doi: 10.1016/j.jmb.2008.06.056 18619468

[38] Umar A, Boland CR, Terdiman JP, Syngal S, de la Chapelle A, Rüschoff J, et al. Revised Bethesda Guidelines for hereditary nonpolyposis colorectal cancer (Lynch syndrome) and microsatellite instability. J Natl Cancer Inst. 18 févr 2004;96(4):261‑8. doi: 10.1093/jnci/djh034 14970275 PMC2933058

[39] Vasen HF, Watson P, Mecklin JP, Lynch HT. New clinical criteria for hereditary nonpolyposis colorectal cancer (HNPCC, Lynch syndrome) proposed by the International Collaborative group on HNPCC. Gastroenterology. juin 1999;116(6):1453‑6. doi: 10.1016/s0016-5085(99)70510-x 10348829

[40] Crooks GE, Hon G, Chandonia JM, Brenner SE. WebLogo: a sequence logo generator. Genome Res. juin 2004;14(6):1188‑90. doi: 10.1101/gr.849004 15173120 PMC419797

[41] Raevaara TE, Korhonen MK, Lohi H, Hampel H, Lynch E, Lönnqvist KE, et al. Functional significance and clinical phenotype of nontruncating mismatch repair variants of MLH1. Gastroenterology. août 2005;129(2):537‑49. doi: 10.1016/j.gastro.2005.06.005 16083711

[42] London J, Martín-López J, Yang I, Liu J, Lee JB, Fishel R. Linker domain function predicts pathogenic MLH1 missense variants. Proc Natl Acad Sci U S A. 2 mars 2021;118(9):e2019215118. doi: 10.1073/pnas.2019215118 33619096 PMC7936337

[43] Worth CL, Blundell TL. On the evolutionary conservation of hydrogen bonds made by buried polar amino acids: the hidden joists, braces and trusses of protein architecture. BMC Evol Biol. 31 mai 2010;10:161. doi: 10.1186/1471-2148-10-161 20513243 PMC2892493

[44] Silva FC da, Ferreira JR de O, Torrezan GT, Figueiredo MCP, Santos ÉMM, Nakagawa WT, et al. Clinical and Molecular Characterization of Brazilian Patients Suspected to Have Lynch Syndrome. PLOS ONE. 5 oct 2015;10(10):e0139753. doi: 10.1371/journal.pone.0139753 26437257 PMC4593564

[45] Comprehensive RNA and protein functional assessments contribute to the clinical interpretation of MSH2 variants causing in-frame splicing alterations | Journal of Medical Genetics [Internet]. [cité 11 févr 2024]. Disponible sur: https://jmg.bmj.com/content/60/5/450.abstract 10.1136/jmg-2022-108576 36113988

[46] Standards and guidelines for the interpretation of sequence variants: a joint consensus recommendation of the American College of Medical Genetics and Genomics and the Association for Molecular Pathology—PubMed. [cité 16 avr 2024]. Disponible sur: https://pubmed.ncbi.nlm.nih.gov/25741868/10.1038/gim.2015.30 PMC4544753 25741868

[47] Hirasawa A, Imoto I, Naruto T, Akahane T, Yamagami W, Nomura H, et al. Prevalence of pathogenic germline variants detected by multigene sequencing in unselected Japanese patients with ovarian cancer. Oncotarget. 22 déc 2017;8(68):112258‑67. doi: 10.18632/oncotarget.22733 29348823 PMC5762508

[48] Pedroni M, Roncari B, Maffei S, Losi L, Scarselli A, Di Gregorio C, et al. A mononucleotide markers panel to identify hMLH1/hMSH2 germline mutations. Dis Markers. 2007;23(3):179–87. doi: 10.1155/2007/703129 17473388 PMC3850839

[49] Mangold E, Pagenstecher C, Friedl W, Mathiak M, Buettner R, Engel C, et al. Spectrum and frequencies of mutations in MSH2 and MLH1 identified in 1,721 German families suspected of hereditary nonpolyposis colorectal cancer. Int J Cancer. 20 sept 2005;116(5):692–702. doi: 10.1002/ijc.20863 15849733

[50] Tournier I, Vezain M, Martins A, Charbonnier F, Baert-Desurmont S, Olschwang S, et al. A large fraction of unclassified variants of the mismatch repair genes MLH1 and MSH2 is associated with splicing defects. Hum Mutat. 2008;29(12):1412–24. doi: 10.1002/humu.20796 18561205

