# Supplementary figures and images for: Functional analysis of MMR gene VUS from potential Lynch syndrome patients
Source: PLoS One. 2024 Jun 6;19(6):e0304141. doi: 10.1371/journal.pone.0304141 (PMC11156341; doi:10.1371/journal.pone.0304141)

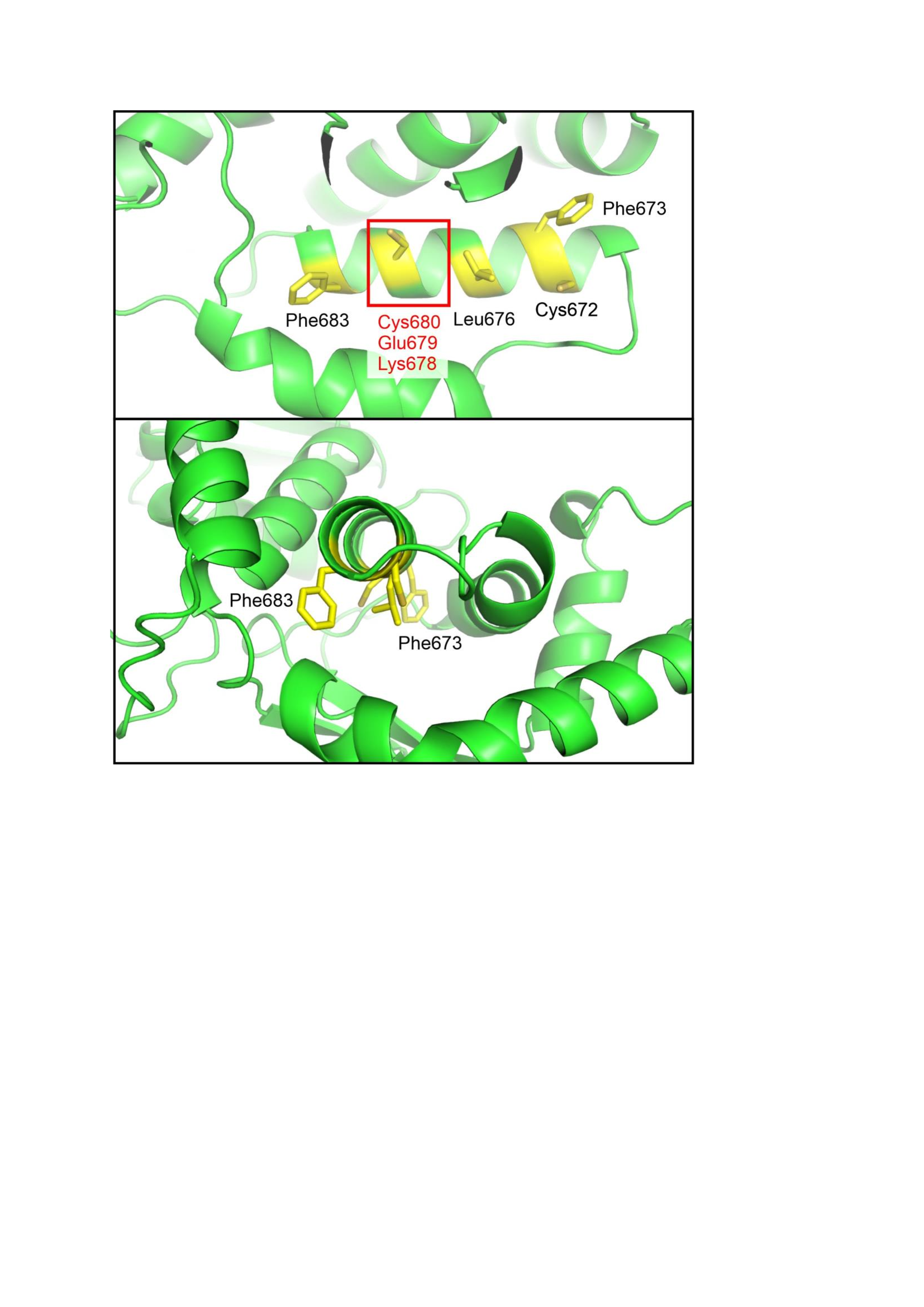

Supplement: S1 Fig — (TIF) [file pone.0304141.s001.tif]
