# Supplementary material for: Functional analysis of MMR gene VUS from potential Lynch syndrome patients
Source: PLoS One. 2024 Jun 6;19(6):e0304141. doi: 10.1371/journal.pone.0304141 (PMC11156341; doi:10.1371/journal.pone.0304141)
Supplement: S1 Table — (DOCX) [file pone.0304141.s002.docx]

# Supporting Material

# Supporting Table 1: ACMG classification for the MMR variants (Guidelines 2015)^1^

***MLH1*** c.541G>A (**p.Gly181Ser)**: **VUS 2 points**, Class 3

| PM1 moderate | Hot-spot of length 17 amino-acids has 30 missense/in-frame variants (4 pathogenic variants, 26 uncertain variants and no benign), which qualifies as moderate pathogenic. |
| --- | --- |
| PM2 supporting | GnomAD genomes homozygous allele count = 0 is less than 2 for AD/AR gene MLH1; gnomAD genomes coverage good (30.5).  Variant not found in gnomAD exomes; gnomAD exomes coverage good (35.0). |
| BP4 supporting | MetaRNN = 0.306 is between 0.267 and 0.43 (supporting benign) |

***MLH1*** c.1013A>G (**p.Asn338Ser)**: **VUS 1 point (Clinvar conflicting)**, Class 3

| PM2 supporting | GnomAD genomes homozygous allele count = 0 is less than 2 for AD/AR gene MLH1; gnomAD genomes coverage good (31.5).  GnomAD exomes homozygous allele count = 0 is less than 2 for AD/AR gene MLH1; gnomAD exomes coverage good (34.7). | |  |
| --- | --- | --- | --- |
| PP5 supporting | UniProt Variants classifies this variant as Pathogenic | |  |
|  |  |  |  |
| BP1 supporting | Out of 1747 pathogenic variants in gene MLH1 there are 213 pathogenic missense variants versus 1534 pathogenic truncating variants. | | |

***MLH1*** c.1940_1951delTGCCCCCTTTGG (**p.Val647_Leu650del**): **Pathogenic, Class 5**

| PM1 very strong | Hot-spot of length 21 amino-acids has 61 missense/in-frame variants (19 pathogenic variants, 42 uncertain variants and no benign), which qualifies as very strong pathogenic.  UniProt protein MLH1_HUMAN region of interest 'Interaction with EXO1' has 652 missense/in-frame variants (71 pathogenic variants, 575 uncertain variants and 6 benign variants), which qualifies as moderate pathogenic. |
| --- | --- |
| PM4 moderate | Protein coding length changes as a result of in frame variant in gene MLH1, and this variant is not located in a repeat region. |
| PM2 supporting | Variant not found in gnomAD genomes; gnomAD genomes coverage good (30.3).  Variant not found in gnomAD exomes; gnomAD exomes coverage good (45.4). |

***MLH1*** c.2031_2039delTAAAGAATG (**p.Lys678_Cys680del**): **Likely Pathogenic, Class 4**

| PM1 strong | Hot-spot of length 20 amino-acids has 61 missense/in-frame variants (8 pathogenic variants, 53 uncertain variants and no benign), which qualifies as strong pathogenic |
| --- | --- |
| PM4 moderate | Protein coding length changes as a result of in frame variant in gene MLH1, and this variant is not located in a repeat region. |
| PM2 supporting | Variant not found in gnomAD genomes, gnomAD genomes coverage good (31.1).  Variant not found in gnomAD exomes, gnomAD exomes coverage good (44.8). |

***MLH1*** c.2180 delA (**p.His727Pro*fs**57**): **VUS 5 points, Class 3**

| PVS1 very strong | Null variant (frame-shift) in gene MLH1, not predicted to cause NMD. Loss-of-function is a known mechanism of disease (gene has 1 534 reported pathogenic LOF variants). The truncated region contains 48 pathogenic variants. It removes 3.96% of the protein. |
| --- | --- |
| PM2 supporting | Variant not found in gnomAD genomes, gnomAD genomes coverage good (31.5).  Variant not found in gnomAD exomes, gnomAD exomes coverage good (31.4). |

***MSH2*** c.2362_2363 insA (**p.Thr788Asn*fs**11**)**: Pathogenic, Class 5**

| PVS1 very strong | Null variant (frame-shift) in gene MSH2, predicted to cause NMD. Loss-of-function is a known mechanism of disease (gene has 1813 reported pathogenic LOF variants). The exon contains 166 pathogenic variants. The truncated region contains 209 pathogenic variants. |
| --- | --- |
| PP5 very strong | ClinVar classifies this variant as pathogenic, 3 stars (expert panel, reviewed Dec 2023, 5 submissions). |
| PM2 supporting | Variant not found in gnomAD genomes, gnomAD genomes coverage good (30.1).  Variant not found in gnomAD exomes, gnomAD exomes coverage good (32.5). |

^1^ Richards S. *et al*.: Standards and guidelines for the interpretation of sequence variants: a joint consensus recommendation of the American College of Medical Genetics and Genomics and the Association for Molecular Pathology. Genet Med. 2015 May;17(5):405-24.
