# Supplementary material for: Functional analysis of MMR gene VUS from potential Lynch syndrome patients
Source: PLoS One. 2024 Jun 6;19(6):e0304141. doi: 10.1371/journal.pone.0304141 (PMC11156341; doi:10.1371/journal.pone.0304141)
Supplement: S1 File — (DOCX) [file pone.0304141.s003.docx]

# Supporting Material

**Supplementary Table 2: Raw data for the analysis of expression of the MLH1 variants**

| Assay number | 1 | 2 | 3 | **Average expression** | Standard deviation |
| --- | --- | --- | --- | --- | --- |
| WT | 100 | 100 | 100 | **100** | 0 |
| G181S | 114 | 89 | 125 | **109** | 18,5 |
| N338S | 150 | 109 | 169 | **142** | 30,7 |
| DV647-L650 | 3 | 3 | 20 | **8** | 9,8 |
| DK678-C680 | 9 | 11 | 11 | **10** | 1,2 |
| A681T | 48 | 13 | 36 | **32** | 17,8 |
| V716M | 96 | 56 | 60 | **70** | 22,0 |

**Supplementary Table 3: Raw data for the analysis of mismatch repair activity of the MLH1 variants**

| Assay number | 1 | 2 | 3 | **Average MMR activity** | Standard Deviation |
| --- | --- | --- | --- | --- | --- |
| EGFP | 14,5 | 35,57 | 22,97 | **24,3** | 10,6 |
| MLH1 wt | 100 | 100 | 100 | **100,0** | 0 |
| G181S | 93,8 | 131,55 | 123,12 | **116,2** | 19,8 |
| N338S | 106,1 | 137,02 | 146,59 | **129,9** | 21,2 |
| DV647-L650 | 49,1 | 49,17 | 31,67 | **43,3** | 10,1 |
| DK678-C680 | 23,7 | 42,75 | 3,78 | **23,4** | 19,5 |
